# Effect of taxifolin on methotrexate-induced oxidative and inflammatory oral mucositis in rats: biochemical and histopathological evaluation

**DOI:** 10.1590/1678-7757-2022-0115

**Published:** 2022-09-19

**Authors:** Zeynep BAYRAMOGLU, Behzad MOKHTARE, Ali Sefa MENDIL, Taha Abdulkadir COBAN, Renad MAMMADOV, Seval BULUT, Zeynep SULEYMAN, Halis SULEYMAN

**Affiliations:** 1 Ataturk University Faculty of Dentistry Department of Oral and Maxillofacial Surgery Erzurum Turkey Ataturk University, Faculty of Dentistry, Department of Oral and Maxillofacial Surgery, Erzurum, Turkey.; 2 Ataturk University Faculty of Veterinary Department of Pathology Erzurum Turkey Ataturk University, Faculty of Veterinary, Department of Pathology, Erzurum, Turkey.; 3 Erciyes University Faculty of Veterinary Department of Pathology Kayseri Turkey Erciyes University, Faculty of Veterinary, Department of Pathology, Kayseri, Turkey.; 4 Erzincan Binali Yildirim University Faculty of Medicine Department of Biochemistry Erzincan Turkey Erzincan Binali Yildirim University, Faculty of Medicine, Department of Biochemistry, Erzincan, Turkey; 5 Erzincan Binali Yildirim University Faculty of Medicine Department of Pharmacology Erzincan Turkey Erzincan Binali Yildirim University, Faculty of Medicine, Department of Pharmacology, Erzincan, Turkey.; 6 Erzincan Binali Yildirim University Faculty of Health Sciences Department of Nursing Erzincan Turkey Erzincan Binali Yildirim University, Faculty of Health Sciences, Department of Nursing, Erzincan, Turkey.

**Keywords:** Taxifolin, Methotrexate, Oral mucositis, Rat

## Abstract

The role of oxidative stress, as well as inflammation in the pathogenesis of methotrexate (MTX)-induced oral mucositis, is a known fact. The anti-inflammatory, antitumor, antimicrobial, and antioxidant properties of taxifolin—the effect we tested against MTX-induced oral mucosal damage—are well known.

**Objective:**

Evaluating biochemically and histopathologically the effects of taxifolin on methotrexate-induced oral mucosal damage in rats.

**Methodology:**

In the taxifolin+MTX (TMTX) group, 50 mg/kg taxifolin was orally administered to rats by gavage. In the MTX and healthy (HG) groups, normal saline was applied to rats as solvent by the same method. One hour after administration of taxifolin and solvent, 5 mg/kg MTX was orally administered to rats in the MTX and TMTX groups. Taxifolin and methotrexate were administered once a day for 30 days. Macroscopic, biochemical, and histopathological evaluations were performed on the inner cheek and tongue tissues of rats. These parts were removed after rats were killed with a high-dose anesthesia.

**Results:**

Taxifolin with MTX prevented the increase in oxidant and pro-inflammatory parameters, such as malondialdehyde (MDA), tumor necrosis factor alpha (TNF-α), interleukin 1 beta (IL-1β), interleukin 6 (IL-6), on the inner cheek and tongue tissues of rats. Moreover, taxifolin antagonized the decrease in total glutathione (tGSH). Taxifolin decreased MTX-induced histopathological damage.

**Conclusion:**

These findings suggest that taxifolin may be useful to treat MTX-associated oral mucositis.

## Introduction

Methotrexate (MTX) is a folic acid antagonist—an antiproliferative drug, as it is known.^1^ Currently, MTX is used in the treatment of pediatric acute lymphoblastic leukemia, choriocarcinoma, and various malignancies. Moreover, MTX is a gold-standard antirheumatic agent in the treatment of rheumatoid arthritis, psoriasis, systemic scleroderma, and other autoimmune disorders.^2^ The use of MTX is preferred in low doses in the treatment of inflammatory diseases while it is preferred in high doses in the treatment of malignant diseases.^1^ As well as high-dose MTX is safely applied to most patients during treatment, serious side effects, such as nephrotoxicity, myelosuppression, mucositis, dermatologic toxicity, and hepatotoxicity, can be observed.^3^ Moreover, various side effects may occur during the treatment of inflammatory diseases, ranging from mild to severe side effects and even leading to treatment discontinuation.^4^ One of the serious side effects of MTX is oral mucositis.^5^ Oral toxicities, such as mucositis and stomatitis, are some of the most serious and indispensable side effects related to cancer therapy.^6^ Mucositis is a pathological process that causes ulceration and inflammation of the mucosal tissue in the region from the mouth to the anus.^7^ Oral mucositis makes feeding difficult and may also increase the risk of infection, limit the dose of the medication, delay the treatment, and extend hospital stay.^8^ The role of reactive oxygen species (ROS) and various pro-inflammatory cytokines in the pathogenesis of mucositis have been shown.^9^ It has also been showed that MTX increases the levels of malondialdehyde (MDA), an oxidant parameter in the oral mucosa, interleukin 1 beta (IL-1β), and tumor necrosis factor alpha (TNF-α), an indicator of inflammatory response.^10^ The literature shows that effective antioxidant, anti-inflammatory, antiulcer, and antimicrobial medication may be useful to treat MTX-associated oral mucositis.

Taxifolin (3, 5, 7, 3, 4-pentahydroxy flavanone), which we tested against MTX-induced oral mucosal damage in this study, can be found in plants such as thistle, onion, French maritime, and tamarind.^11^ The anti-inflammatory, antitumor, antimicrobial, and antioxidant properties of taxifolin are well known.^12^ It has been shown that taxifolin has an antioxidant effect by inhibiting ROS production.^13^ The antioxidant, anti-inflammatory, and antimicrobial properties of taxifolin suggest that it may be useful to treat MTX-induced oral mucosal damage. In the literature, the oxidative and inflammatory effects of taxifolin associated with MTX have been reported. There are no studies in the literature evaluating the protective effect of taxifolin against MTX-induced oxidative and inflammatory mucosal damage. Thus, our study aimed to evaluate biochemically and histopathologically the effects of taxifolin on MTX-induced oxidative and inflammatory oral mucosal damage in rats.

## Methodology

### Rats

Male albino Wistar rats were obtained from Erzincan Binali Yildirim University Experimental Animals Application and Research Center. In the study, 18 healthy rats weighing from 280 to 295 g were used. Rats were fed and separated in groups (n=6) in the suitable laboratory environment at normal room temperature (22°C) before the experiment. Procedures and protocols were approved by the Erzincan Binali Yildirim University Ethics Committee for Animal Experimentation on January 27, 2022 (Decision no. 2).

### Chemicals

The chemicals used were methotrexate, thiopental sodium, and taxifolin and they were obtained from Med-İlaç, Turkey, I.E. Ulagay, Turkey, and Evalar, Russia, respectively.

### Experimental groups

The rats used in the experiment were divided into three groups: MTX group , taxifolin+MTX group (TMTX), and healthy group (HG).

### Experiment procedure

A total of 50 mg/kg taxifolin^14^ was administered orally to the stomach of rats by gavage in the TMTX group (n=6). Normal saline (0.9% NaCl) was applied to rats in MTX (n=6) and healthy (n=6) groups as solvent by the same method. One hour after taxifolin and solvent administration, animals in the MTX and TMTX groups orally received 5 mg/kg MTXo.^15^ Taxifolin and methotrexate were administered once a day for 30 days. At the end of this period, all rats were killed with high-dose anesthesia (50 mg/kg thiopental sodium). The inner cheek and tongue tissues were removed and macroscopically evaluated, and then the levels of MDA, tGSH, NF-kB, TNF-α, IL-1β, and IL-6 were determined from the tissues. Tissues were also analyzed histopathologically. The results of the experiments in all groups were compared between groups and evaluated.

### Tissue MDA and tGSH determination

Tissue MDA (µmol/g protein) was measured by the method used by Ohkawa, Ohishi and Yagi^16^ (1979). On the other hand, tGSH (nmol/g protein) was measured by the method described by Sedlak J and Lindsay RH.^17^

### Tissue TNF-α, IL-1β, and IL-6 analysis

The samples were weighed and then the tissues were cut, frozen with liquid nitrogen, and homogenized with a pestle and mortar. The samples were maintained at 2°C to 8°C after melting. Phosphate buffered saline (PBS) (pH 7.4; 1/10 w/v) was added and, after vortex for 10 seconds, centrifuged at 10000 xg for 20 minutes, the supernatants were carefully collected. The levels of TNF-α (ng/L), IL-1β (pg/L), and IL-6 (ng/L) were measured according to a commercial kit supplied by ELISA kit, Eastbiopharm Co., Ltd., China.

### Histopathological analyses

The inner cheek and tongue tissues were fixed in 10% neutral formalin solution. The tissues were subjected to routine alcohol-xylol series after fixation. Later, 5 µm sections of the sample were placed in paraffin blocks and six random areas were stained with hematoxylin and eosin. Sections were stained with hematoxylin and eosin. The evaluation was made according to Table 1: normal (-), mild (+), moderate (++), and severe (+++) damage.

**Table 1:** Histopathological scores

Tongue			Inner cheek		
Keratinized papillae	< 10%	+++	Inflammatory cell infiltration	none	-
	10–40% 40–70%	++ +		1–3% 3–5%	+ ++
	> 70%	-		> 5%	+++
Epithelial thickness/cell layer	0–1	+++	Hemorrhage	none	-
	1–3 3–5	++ +		1–10% 10–20%	+ ++
	> 5	-		> 20%	+++
Swelling in the lamina propria	< 10%	-	Muscle fiber degeneration	none	-
	10–30%	+		1–5%	+
	30–50%	++		5–10%	++
	> 50%	+++		> 10%	+++

For biochemical analysis

### Statistical analysis

For biochemical analysis, the Shapiro-Wilk test was used to determine whether data were normally distributed. Since data were normally distributed, the evaluation was made by one-way ANOVA. The Tukey or Games-Howell test was performed according to the results of the Levene test as a post hoc test. Biochemical results are presented as mean±standard deviation. Since histopathological data are ordinal data, the evaluation was made with the Mann-Whitney U test. Histopathological results are presented as mean±standard deviation and median (minimum–maximum). All statistical procedures were performed with IBM SPSS 22.0 (IBM Corp., Armonk, NY) and p<0.05 was considered significant.

## Results

### Results of the macroscopic evaluation of the inner cheek and tongue tissues

**Figure 1:**
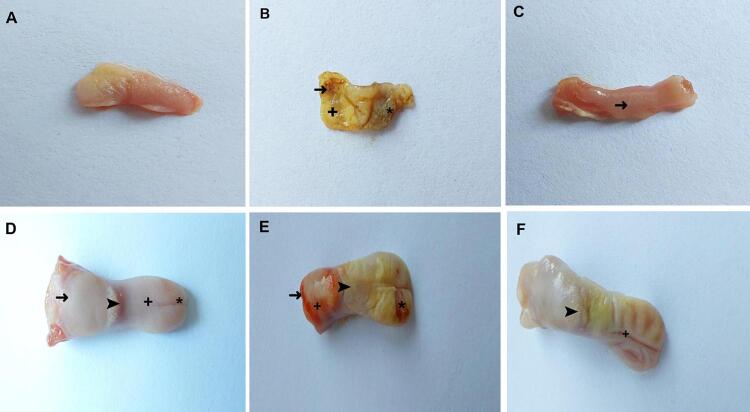
Macroscopic evaluation of the inner cheek and tongue tissues in the groups.

In the MTX group, the inner cheek tissue was quite swollen and presented severe hemorrhage, hyperemia, and ulcerated areas. In the TMTX group, the inner cheek tissue was less swollen and there was moderate swelling and hyperemia. When the tongue tissue was macroscopically evaluated, hyperemic muscle—due to the loss of the full-thickness epithelial layer on the tip of the tongues of the MTX group—complete loss of papillae, significant swelling of the entire tongue, hemorrhage, and hyperemia were observed. In the tongues of the TMTX group, moderate hyperemia was observed in the body and tip of the tongues, with a decrease in the papillary prominence (Figure 1).

A. Normal healthy inner cheek tissue of the healthy group. B. Inner cheek tissue of the MTX group. Swelling (→); severe hemorrhage and hyperemia (+); ulcerated areas (*). C. Inner cheek tissue of the TMTX group. Moderate swelling and hyperemia (→) D. Normal tongue tissue of the healthy group. Tongue body (+); tongue tip (*); tongue root (→); papillae (➤). E. Tongue tissue of the MTX group. The entire tongue is swollen; muscle hyperemia due to loss of the full-thickness epithelial layer on the tip (*); complete loss of papillae (➤); hemorrhage (→); hyperemia in the root (+). F. Tongue tissue of the TMTX group. Moderate swelling; indistinct papillae (➤); moderate hyperemia on the body and tip (+)

## Biochemical findings

### Results of the MDA analysis in the inner cheek and tongue tissues

The amount of MDA in the inner cheek and tongue tissues of rats treated with MTX increased significantly when compared to the HG, as it can be observed in Figure 2 (p<0.001). The increase in MDA was significantly inhibited by taxifolin (p<0.001). There was a significant difference between the healthy and TMTX groups in terms of MDA values in the inner check tissue (p=0.036), but there was no significant difference between samples in the tongue tissue (p=0.630).

### Results of the tGSH analysis in the inner cheek and tongue tissues

As it can be seen in Figure 2, the amount of tGSH in the inner cheek and tongue tissues of rats treated with MTX was lower than that of the HG (p<0.001). Taxifolin significantly inhibited the reduction of tGSH in the inner cheek and tongue tissues of rats receiving MTX <0.001 (Figure 2).

### Results of the TNF-α analysis in the inner cheek and tongue tissues

As it can be seen in Figure 2, the amount of TNF-α in the inner cheek and tongue tissues of rats treated with MTX was higher than that of the HG (p<0.001). Taxifolin significantly inhibited the reduction of TNF-α in the inner cheek and tongue tissues of rats receiving MTX (p<0.001).

### Results of the IL-1β analysis in the inner cheek and tongue tissues

MTX increased the amount of IL-1β in the inner cheek and tongue tissues of rats (p<0.001). However, taxifolin significantly inhibited the increase in IL-1β in rats receiving MTX (p<0.001) (Figure 2).

### Results of the IL-6 analysis in the inner cheek and tongue tissues

The amount of IL-6 in the inner cheek and tongue tissues of rats treated with MTX increased significantly when compared to the HG, as it can be observed in Figure 2 (p<0.001). Taxifolin significantly inhibited the increase in IL-6 in the inner cheek and tongue tissues of rats receiving MTX (p<0.001). Statistical analyses showed that the difference in the amount of IL-6 between the TMTX and healthy groups was insignificant (p>0.05) (Figure 5).

## Histopathological findings

The histopathological findings were higher in the inner cheek and tongue tissues of rats in the MTX group than in the TMTX group. Statistically significant differences were observed between groups in terms of histopathological findings (Table 3; p<0.05).

**Table 3:** Histopathological damage classification

			Groups		
	Findings	Median (minimum–maximum)			
		HG	MTXG	TMTX	p-values*
Tongue	Keratinized papillae	0 _(0–0)_	2 _(2–3)_	1 _(0–2)_	0.041
	Epithelial thickness	0 _(0–0)_	2 _(2–2)_	1 _(1–2)_	0.015
	Swelling in the lamina propria	0 _(0–0)_	2 _(1–2)_	2 _(1–2)_	0.699

Inner cheek	Inflammatory cell infiltration	0 _(0–0)_	3 _(2–3)_	1 _(0–1)_	0.002
	Hemorrhage	0 _(0–0)_	3 _(2–3)_	1 _(0–1)_	0.002
	Muscle fiber degeneration	0 _(0–0)_	3 _(1–3)_	1 _(0–1)_	0.002

0: normal; 1: mild damage; 2: medium damage; 3: severe damage. HG: healthy group; MTXG: methotrexate group; TMTX: taxifolin+methotrexate group. *Statistical analysis was performed between the MTXG and TMTX groups by the Mann-Whitney U test.

**Figure 3:**
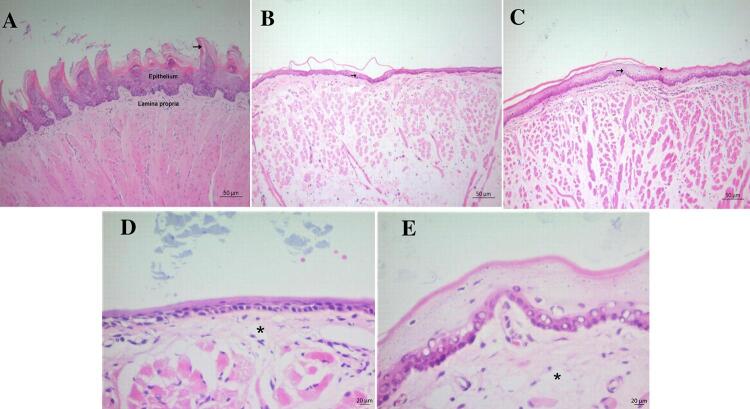
Histopathological evaluation of the tongue tissue in the groups.

The tongue tissue presented a normal histological appearance in rats in the HG (Figure 3A). Keratinized papillae were slightly shaped in the TMTX group while no keratinized papillae were observed in the tongues of the MTX group. The reduction in epithelial thickness was moderate in the MTX group and mild in the TMTX group. Swelling was moderate in the MTX and TMTX groups (Figure 3B-E).

**Figure 4:**
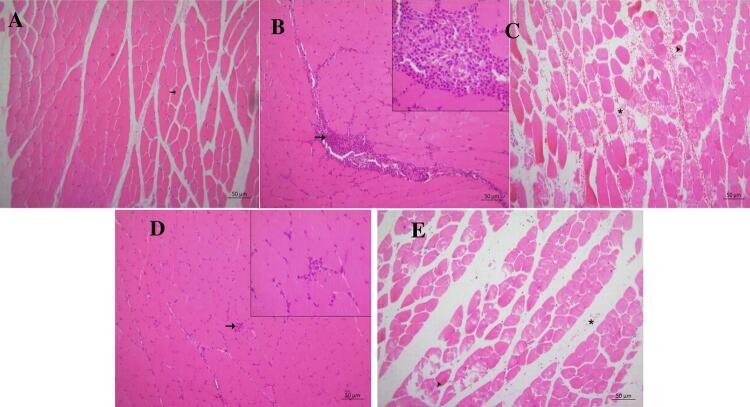
Histopathological evaluation of the inner cheek tissue in the groups.

The inner cheek tissue presented a normal histological appearance in rats in the HG (Figure 4A). Interstitial inflammatory cell infiltration and hemorrhage were severe in the MTX group while these histopathological findings were mild in the TMTX group. Similarly, muscle fiber degeneration was severe in the MTX group and mild in the TMTX group (Figure 4B-E).

## Discussion

The effects of taxifolin on MTX-induced oxidative and inflammatory oral mucosal damage in rats were evaluated macroscopically, biochemically, and histopathologically in this study. Our macroscopic findings showed that mucositis developed in the inner cheek and tongue tissues of rats treated with MTX. Our biochemical findings showed that MTX increased MDA, TNF-α, IL-1β, and IL-6, and decreased tGSH in the inner cheek and tongue tissues. MTX also caused histopathological damage. Taxifolin significantly prevented macroscopic, biochemical, and histopathological changes.

A. Tongue tissue of the healthy group. Normal histological appearance with keratinized papillae (arrow) (HxE). B. Tongue tissue of the MTX group. Thin epithelial thickness (arrow) (HxE). C. Tongue tissue of the TMTX group. Moderate epithelial thickness (arrow) and keratinized papillae (arrowhead) (HxE). D. Tongue tissue of the MTX group. Moderate swelling (*) in the lamina propria (HxE). E. Tongue tissue of the TMTX group. Moderate swelling (*) in the lamina propria. (HxE).

A. Inner cheek tissue of the healthy group. Normal histological appearance (arrow) of muscles and fibers (HxE). B. Inner cheek tissue of the MTXG group. Severe inflammatory cell infiltration (arrow) in interstitial areas (HxE). C. Inner cheek tissue of the MTX group. Severe hemorrhage (*) and appearance of degenerative muscle fibers (arrowhead) in interstitial areas (HxE). D. Buccal tissue belonging to the TMTX group. Mild inflammatory cell infiltration (arrow) appearance in interstitial areas. (HxE). E. Buccal tissue belonging to the TMTX group. Mild hemorrhage (*) in interstitial areas and muscle fiber degeneration (arrowhead) (HxE).

**Table 2:** Results of the biochemical analysis of the inner cheek and tongue tissues

Biochemical		Mean±standard deviation				p-values	
parameters		HG	MTXG	TMTX	HG vs. MTXG	HG vs. TMTX	MTXG vs. TMTX
Inner cheek	MDA	3.32±0.11	6.14±0.20	3.56±0.12	<0.001	0.036	<0.001
	tGSH	5.67±0.13	2.54±0.22	5.10±0.22	<0.001	<0.001	<0.001
	TNF-α	2.13±0.08	5.09±0.25	2.44±0.08	<0.001	<0.001	<0.001
	IL-1β	1.26±0.07	3.58±0.35	1.52±0.14	<0.001	0.011	<0.001
	IL-6	1.73±0.07	3.92±0.20	1.83±0.20	<0.001	0.432	<0.001

Tongue	MDA	4.13±0.58	6.88±0.16	4.37±0.23	<0.001	0.630	<0.001
	tGSH	5.34±0.16	2.20±0.12	4.76±0.19	<0.001	<0.001	<0.001
	TNF-α	2.29±0.10	4.70±0.95	2.45±0.08	<0.001	0.020	<0.001
	IL-1β	1.42±0.07	3.81±0.09	1.65±0.09	<0.001	0.001	<0.001
	IL-6	2.02±0.24	4.20±0.31	2.22±0.19	<0.001	0.374	<0.001

MDA: malondialdehyde; tGSH: total glutathione; TNF-α: tumor necrosis factor alpha; IL-1β: interleukin 1 beta; IL- 6: interleukin 6: HG: healthy group; MTXG: methotrexate group: TMTX: taxifolin+methotrexate group. The analysis was performed by one-way ANOVA and the Tukey or Games-Howell test was used as a post hoc test.

Oral mucositis is one of the serious side effects of MTX, as aforementioned.^5^ Mucositis is a pathological process characterized by ulceration and inflammation of the mucosal tissue.^18^ Another symptom of mucositis is bleeding of the mucosa.^19^ In our study, symptoms such as swelling, hyperemia, ulcer, and hemorrhage were observed in the inner cheek and tongue tissues of rats treated with MTX, which is in accordance with the literature.

The role of ROS and various pro-inflammatory cytokines in the pathogenesis of mucositis have been shown in the literature.^9^ The levels of MDA, tGSH, TNF-α, IL-1β, and IL-6 were measured in the inner cheek and tongue tissues in order to evaluate MTX-induced mucositis. Our biochemical findings showed that MTX increased MDA and decreased tGSH in the inner cheek and tongue tissues. These findings show that oxidative stress developed in the inner cheek and tongue tissues of rats treated with MTX. Oxidant increase and antioxidant decrease in living tissues are also considered oxidative damage in the literature.^20^ Our biochemical findings are in accordance with studies by Kuduban, et al.^10^ (2016), which showed that MTX increases MDA and decreases GSH in the oral mucosa. It is known that MTX changes the balance between MDA and tGSH in favor of MDA in other tissues besides the oral mucosa.^21^ MDA is the cytotoxic product of the lipid peroxidation (LPO) reaction initiated by ROS,^22,23^ as it is known. Thus, MDA became one of the most popular and reliable markers of oxidative stress nowadays.^24^

In our study, the amount of MDA in the TMTX group was close to that of the HG. These findings suggest that taxifolin is supported by studies that show its inhibitory effect on LPO and ROS production.^13,25^ Taxifolin has also been reported to have a scavenging effect on other ROS, especially hydroxyl radical (HO).^26^ The fact that taxifolin has an inhibitory effect on ROS^27^ can be considered as its mechanism to prevent MTX from decreasing tGSH in the oral mucosa. These findings suggest that tGSH was insufficient to neutralize ROS in the MTX group. GSH is a tripeptide consisting of L-glutamate, L-cysteine, and glycine that found in many cells, as it is known.^28^ GSH, when catalyzed by selenium-glutathione peroxidase, reacts with hydrogen peroxide (H_2_O_2_) and chemically detoxifies H_2_O_2_ and protects cells from ROS damage.^22,29^

Our biochemical findings showed that MDA, TNF-α, IL-1β, and IL-6 increased and tGSH decreased in the MTX group. In a study that support our findings, it was reported that MTX increased the levels of MDA, IL-1β and TNF-α in the oral mucosa.^10^ Our experimental results and this information obtained from the literature point to the relationship between oxidative stress and inflammation. It has been reported in the literature that ROS, which cause oxidative stress, play an important role in the regulation of inflammation.^30^ Moreover, it has been stated that mitochondrial ROS are signaling molecules that trigger pro-inflammatory cytokine production.^31^ It was reported that GSH and enzymatic antioxidant levels decreased in cells with increased levels of TNF-α, IL-1β, IL-6, and LPO in a study evaluating the relationship between oxidant and pro-inflammatory cytokine.^32^ It was shown that antioxidants excessively produced ROS and reduced inflammatory responses in another study.^30^ Taxifolin with MTX significantly prevented the increase in TNF-α, IL-1β, and IL-6 in our study, which overlaps is in accordance with the literature. There were no studies evaluating the effects of taxifolin on TNF-α, IL-1β, and IL-6 in the inner cheek and tongue tissues of rats treated with MTX. However, the nephroprotective effect of taxifolin of inhibiting the increase in MDA, TNF-α, and IL-1β in the kidneys with acrylamide is a known fact.^33^ Inhibition of IL-6 is also involved in the pathogenesis of the anti-inflammatory effect of taxifolin.^34^

Our biochemical findings, obtained from inner cheek and tongue tissue samples, were in accordance with the histopathological findings in our study. Papillary structures disappeared, epithelial thickness decreased, and there was swelling in the tongues of rats treated with MTX. These histopathological findings are also in accordance with previous studies.^35^ Moreover, MTX caused inflammatory cell infiltration, hemorrhage, and muscle fiber degeneration in the inner cheek tissue. Inflammatory cell infiltration was observed in the inner cheek of rats treated with MTX in a study by Bilginaylar, et al.^36^ (2022). Furthermore, it has been reported that MTX produces degenerative changes in the epithelial cells of the inner cheek and tongue surface.^37^ The fact that taxifolin alleviated histopathological damage in the inner cheek and tongue tissues due to MTX may be due to its antioxidant and anti-inflammatory effects. The role of ROS and various pro-inflammatory cytokines in the pathogenesis of mucositis has been shown in the literature.^9^

## Conclusion

The development of oxidative and inflammatory damage in the inner cheek and tongue tissues of rats treated with MTX was evaluated biochemically and histopathologically. Taxifolin antagonized MTX-induced oxidant and pro-inflammatory cytokines increased and the antioxidant property decreased in the inner cheek and tongue tissues. Taxifolin also reduced the histopathological damage associated with MTX administration in the inner cheek and tongue tissues. Our biochemical and histopathological findings suggest that taxifolin may be useful in the treatment of MTX-induced oral mucositis. More detailed studies are needed in the future to clarify the protective effect of taxifolin against MTX-induced oxidative and inflammatory oral mucositis.

**Figure 2:**
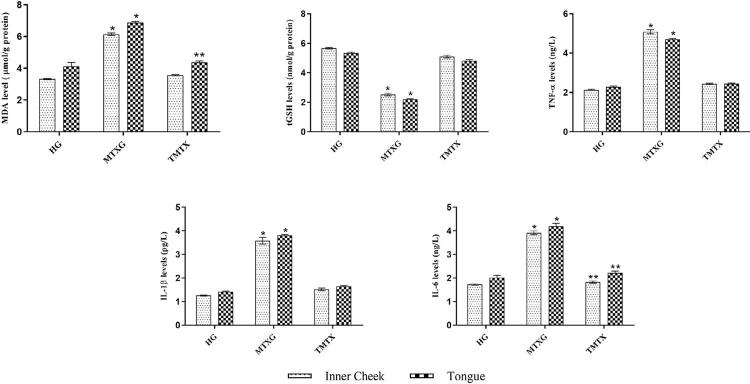
MDA, tGSH, TNF-α, IL-1β, and IL-6 analyses in the inner cheek and tongue tissues of the groups.

*p<0.001, according to the healthy and TMTX groups. **p>0.05, according to the healthy group. MDA: malondialdehyde; tGSH: total glutathione; TNF-α: tumor necrosis factor alpha; IL-1β: interleukin 1 beta; IL-6: interleukin 6; HG: healthy group: MTXG: methotrexate group; TMTX: taxifolin+methotrexate group

## References

[1] Maksimovic V, Pavlovic-Popovic Z, Vukmirovic S, Cvejic J, Mooranian A, Al-Salami H (2020). Molecular mechanism of action and pharmacokinetic properties of methotrexate. Mol Biol Rep.

[2] Řiháček M, Pilatova K, Štěrba J, Pilný R, Valík D (2015). Nové poznatky ve farmakologii methotrexátu: diagnostické možnosti a klinický význam [New findings in methotrexate pharmacology: diagnostic possibilities and impact on clinical care]. Klin Onkol.

[3] Howard SC, McCormick J, Pui CH, Buddington RK, Harvey RD (2016). Preventing and managing toxicities of high-dose methotrexate. Oncologist.

[4] Wang W, Zhou H, Liu L (2018). Side effects of methotrexate therapy for rheumatoid arthritis: a systematic review. Eur J Med Chem.

[5] Maiguma T, Hayashi Y, Ueshima S, Kaji H, Egawa T, Chayama K (2008). Relationship between oral mucositis and high-dose methotrexate therapy in pediatric acute lymphoblastic leukemia. Int J Clin Pharmacol Ther.

[6] Epstein JB, Thariat J, Bensadoun RJ, Barasch A, Murphy BA, Kolnick L (2012). Oral complications of cancer and cancer therapy: from cancer treatment to survivorship. CA Cancer J Clin.

[7] Sema Y, Kenan D, İbrahim B, Can A, Gulsum U, Cigdem E (2012). The effectiveness of grape extract in rats with methotrexate induced intestinal mucositis. J Med Sci.

[8] Curra M, Gabriel AF, Ferreira MB, Martins MA, Brunetto AT, Gregianin LJ (2021). Incidence and risk factors for oral mucositis in pediatric patients receiving chemotherapy. Support Care Cancer.

[9] Sonis ST, Elting LS, Keefe D, Peterson DE, Schubert M, Hauer-Jensen M (2004). Perspectives on cancer therapy-induced mucosal injury: pathogenesis, measurement, epidemiology, and consequences for patients. Cancer.

[10] Kuduban O, Mazlumoglu MR, Kuduban SD, Erhan E, Cetin N, Kukula O (2016). The effect of hippophae rhamnoides extract on oral mucositis induced in rats with methotrexate. J Appl Oral Sci.

[11] Thuan NH, Shrestha A, Trung NT, Tatipamula VB, Van Cuong D, Canh NX (2022). Advances in biochemistry and the biotechnological production of taxifolin and its derivatives. Biotechnol Appl Biochem.

[12] Topal F, Nar M, Gocer H, Kalin P, Kocyigit UM, Gülçin İ (2016). Antioxidant activity of taxifolin: an activity-structure relationship. J Enzyme Inhib Med Chem.

[13] Cai C, Liu C, Zhao L, Liu H, Li W, Guan H (2018). Effects of taxifolin on osteoclastogenesis in vitro and in vivo. Front Pharmacol.

[14] Akagunduz B, Ozer M, Ozcıcek F, Kara AV, Lacın S, Özkaraca M (2021). Protective effects of taxifolin on pazopanib-induced liver toxicity: an experimental rat model. Exp Anim.

[15] Kuduban O, Mazlumoglu MR, Kuduban SD, Erhan E, Cetin N, Kukula O (2016). The effect of hippophae rhamnoides extract on oral mucositis induced in rats with methotrexate. J Appl Oral Sci.

[16] Ohkawa H, Ohishi N, Yagi K (1979). Assay for lipid peroxides in animal tissues by thiobarbituric acid reaction. Anal Biochem.

[17] Sedlak J, Lindsay RH (1968). Estimation of total, protein-bound, and nonprotein sulfhydryl groups in tissue with Ellman’s reagent. Anal Biochem.

[18] Troeltzsch M, von Blohn G, Kriegelstein S, Woodlock T, Gassling V, Berndt R (2013). Oral mucositis in patients receiving low-dose methotrexate therapy for rheumatoid arthritis: report of 2 cases and literature review. Oral Surg Oral Med Oral Pathol Oral Radiol.

[19] Karton E, Vakushina E, Seleskeridi V, Grigorenko M (2020). Improvement of methods for the prevention and hygiene of the oral mucosa of stomathological patients undergoing high dosage chemotherapy. Georgian Med News.

[20] Kisaoglu A, Borekci B, Yapca OE, Bilen H, Suleyman H (2013). Tissue damage and oxidant/antioxidant balance. Eurasian J Med.

[21] Demiryilmaz I, Sener E, Cetin N, Altuner D, Suleyman B, Albayrak F (2012). Biochemically and histopathologically comparative review of thiamine’s and thiamine pyrophosphate’s oxidative stress effects generated with methotrexate in rat liver. Med Sci Monit.

[22] Süleyman H, Özçiçek A (2020). Molecular mechanism of ischemia reperfusion injury. Arch Basic Clin Res.

[23] Giera M, Lingeman H, Niessen WM (2012). Recent advancements in the LC- and GC-based analysis of malondialdehyde (MDA): a brief overview. Chromatographia.

[24] Ayala A, Muñoz MF, Argüelles S (2014). Lipid peroxidation: production, metabolism, and signaling mechanisms of malondialdehyde and 4-hydroxy-2-nonenal. Oxid Med Cell Longev.

[25] Yun BS, Lee IK, Kim JP, Chung SH, Shim GS, Yoo ID (2000). Lipid peroxidation inhibitory activity of some constituents isolated from the stem bark of Eucalyptus globulus. Arch Pharm Res.

[26] Li X, Xie H, Jiang Q, Wei G, Lin L, Li C (2017). The mechanism of (+) taxifolin’s protective antioxidant effect for •OH-treated bone marrow-derived mesenchymal stem cells. Cell Mol Biol Lett.

[27] Ye Y, Wang X, Cai Q, Zhuang J, Tan X, He W, Zhao M (2017). Protective effect of taxifolin on H2O2-induced H9C2 cell pyroptosis. Zhong Nan Da Xue Bao Yi Xue Ban.

[28] Owen JB, Butterfield DA (2010). Measurement of oxidized/reduced glutathione ratio. Methods Mol Biol.

[29] Brigelius-Flohé R, Maiorino M (2013). Glutathione peroxidases. Biochim Biophys Acta.

[30] Sho T, Xu J (2019). Role and mechanism of ROS scavengers in alleviating NLRP3-mediated inflammation. Biotechnol Appl Biochem.

[31] Naik E, Dixit VM (2011). Mitochondrial reactive oxygen species drive proinflammatory cytokine production. J Exp Med.

[32] Wu L, Pan Y (2019). Reactive oxygen species mediate TNF-α-induced inflammatory response in bone marrow mesenchymal cells. Iran J Basic Med Sci.

[33] Bedir F, Kocatürk H, Yapanoğlu T, Gürsul C, Arslan R, Mammadov R (2021). Protective effect of taxifolin against prooxidant and proinflammatory kidney damage associated with acrylamide in rats. Biomed Pharmacother.

[34] Hou J, Hu M, Zhang L, Gao Y, Ma L, Xu Q (2020). Dietary taxifolin protects against dextran sulfate sodium-induced colitis via NF-κB signaling, enhancing intestinal barrier and modulating gut microbiota. Front Immunol.

[35] Al-Moula AD, Ahmed MH, Ahmed MH (2020). Histological study on the effect of methotrexate on the oral tissues of adult male rabbits. Al-Rafidain Dental Journal.

[36] Bilginaylar K, Aykac A, Sayiner S, Özkayalar H, Şehirli AÖ (2022). Evaluation of the antiapoptotic and anti-inflammatory properties of chitosan in methotrexate-induced oral mucositis in rats. Mol Biol Rep.

[37] Ahmed AA, Selim MA, El-Sayed NM (2017). α-Lipoic acid ameliorates oral mucositis and oxidative stress induced by methotrexate in rats: histological and immunohistochemical study. Life Sci.

